# Anterior Cruciate Ligament Reconstruction Return-to-Sport Decision-Making: A Scoping Review

**DOI:** 10.1177/19417381221147524

**Published:** 2023-01-27

**Authors:** Eric Golberg, Mark Sommerfeldt, Adam Pinkoski, Liz Dennett, Lauren Beaupre

**Affiliations:** † Physical Therapy, Faculty of Rehabilitation Medicine, University of Alberta, Edmonton, Canada; ‡ Division of Orthopedic Surgery, Department of Surgery, Faculty of Medicine and Dentistry, University of Alberta, Edmonton, Canada; § Glen Sather Sports Medicine Clinic, University of Alberta, Edmonton, Canada; ‖ Epidemiology, School of Public Health, University of Alberta, Edmonton, Canada; ¶ Scott Health Sciences Library, Faculty of Medicine and Dentistry, University of Alberta, Edmonton, Alberta, Canada

**Keywords:** anterior cruciate ligament, athletic performance assessment, return to sport

## Abstract

**Context:**

Clinical guidelines support the use of testing batteries to assess athlete readiness for return to sport (RTS) and risk of reinjury after anterior cruciate ligament (ACL) reconstruction (ACL-R). There is no consensus on the composition of the testing batteries. Test selection is based mainly on commonality in research, personal preference, and equipment availability. Including athletic performance assessments (APA) used in the athlete’s sport may assist RTS decision-making for stakeholders.

**Objective:**

To determine whether APA for speed, agility, strength, or cardiovascular endurance are (1) used in ACL-R RTS literature and (2) indicative of RTS or reinjury rates.

**Data Sources:**

A systematic search was performed in MEDLINE, EMBASE, CINAHL, SPORTDiscus, Scopus, Web of Science, and ProQuest Dissertations and Theses Global.

**Study Selection:**

Eligibility criteria were as follows: (1) athletes between 6 months and 2 years post-ACL-R, (2) commonly used APA, (3) peer-reviewed primary study with original published data.

**Study Design:**

Scoping Review.

**Level of Evidence:**

Level 4.

**Data Extraction:**

A total of 17 studies included 24 instances of APA with a high degree of heterogeneity for both tests and protocols.

**Results:**

Agility makes up 75% of the APA. Only 17.6% of studies reported RTS or reinjury rates, none of which reported a significant relationship between these rates and APA outcomes.

**Conclusion:**

Speed, strength, and cardiovascular endurance tests are underrepresented in ACL-R RTS literature. Compared with healthy controls, deficits in APA results for ACL-R athletes were common; however, many studies reported significant improvements in results for ACL-R athletes over time. There is some evidence that well-trained ACL-R athletes can match the performance of uninjured athletes in high-level sports.

Anterior cruciate ligament (ACL) tears are among the most common knee ligament injuries in sport.^7,41^ Treatment often involves reconstruction surgery (ACL-R) and lengthy rehabilitation.^41^ Rates of ACL tears are exceptionally high among adolescent and amateur athletes.^8,30,41,52^ The ACL injury incidence rates are 1 of 50 male and 1 of 36 female athletes throughout 1 season.^30^ The impact this injury can have on an athletic career can be devastating, as only about 40% to 60% of athletes can return to the same level of sports competition.^3,37^ Furthermore, the likelihood of reinjury of the ipsilateral or contralateral ACL is 19.4% if the athlete returns to their sport 9 months postsurgery and 7 times greater for those who return earlier.^5,18^ These alarming statistics have created a demand from stakeholders, including surgeons, practitioners, patients, and coaches, for valid and reliable return-to-sport (RTS) practices and protocols, including testing or monitoring of biomotor abilities.

Several highly appraised clinical practice guidelines help stakeholders navigate the ACL-R rehabilitation and RTS process.^1^ Despite somewhat vague recommendations, clinical practice guidelines are a good starting point for improving ACL-R RTS success rates. Each guideline recommends a multidisciplinary approach to ACL-R rehabilitation and RTS criteria; however, there is no clear consensus for RTS testing nor is there substantial evidence that any specific RTS test or battery of tests can predict the risk of reinjury better than others.^1,12,45^ Still, there are promising outcomes from using RTS testing batteries.^11,18,45^ A clear understanding of which RTS tests should make up a testing battery tailored to the patient’s demographics would benefit all stakeholders.

Many different criteria have been used to determine readiness for RTS.^10,45^ Time from surgery is the most prevalent criterion, represented in approximately 85% of the published literature.^10^ In addition, time from surgery was the only criterion in 42% of the included studies.^10^ Strength, the second most common criterion, was represented in 41% of the RTS testing research.^10^ Of studies reporting quantitative strength measures, leg symmetry index (LSI) appeared to be the primary variable of interest rather than absolute or relative strength values.^10^ Hop test criteria were reported in 14% of the reviewed literature; again, LSI appears to be the primary variable of interest, and benchmarks in absolute values were lacking.^10^ Performance-based criteria occurred in 20% of the ACL-R RTS research; however, only a marginal number of reviewed studies (2.9%) specifically utilized APA.^10^ Due to the context-specific demands of sports, it seems beneficial for athletes to undergo several APA to assess their functional ability and fitness levels to be cleared for RTS.^9^

This scoping review aimed to determine which APA (speed, agility, strength, and cardiovascular endurance) have been incorporated into the RTS process. It secondarily examined whether the APA outcomes inform RTS decision-making and whether these tests predict RTS or ACL reinjury rates. The APA protocols and other descriptive variables such as age, sex, sport, and competitive level were reported. The comparison of results between ACL-R athletes, healthy control groups (HCG), benchmarks, or normative data was also reported.

## Methods

A scoping review was chosen due to the broad nature of the research question and expanded inclusion criteria compared with a traditional systematic review.^31^ A scoping review is advantageous because it explores, summarizes, and disseminates research findings and identifies existing literature gaps.^4^ This review follows the 5-stage methodological framework of Arksey and O’Malley^4^ and guidance from the Joanna Briggs Institute Reviewer Manual.^35^ The Preferred Reporting Items for Systematic Reviews and Meta-Analyses (PRISMA) Extension for Scoping Reviews (PRISMA-ScR) was chosen to conduct and report this review.^44^

### Eligibility Criteria

Definitions of APA can vary greatly depending on their context. Therefore, this review focused on physical fitness assessments of speed, agility, strength, and cardiovascular endurance; assessments commonly used in sports settings such as collegiate or professional sports draft combine fitness testing events. Tests used solely in the context of ACL-R RTS were not included. The constructs of “athletic performance assessment”, “speed”, “agility”, “strength”, and “cardiovascular endurance” are operationalized in Table 1. The complete inclusion and exclusion criteria are reported in Table 2.

**Table 1.** Definitions

Construct	Definition
Athletic performance assessment	The quantified representation of an athlete’s biomotor abilities, such as strength, speed, agility, or cardiovascular endurance.
Strength	The ability of an athlete to carry out work against resistance. Tests of maximal load lifted successfully through a predetermined range of motion for predetermined repetitions indicate the maximal force an athlete can generate.^50^
Speed	The ability of an athlete to accelerate from a stationary position and run linearly, covering a set distance in the quickest time possible.^51^
Agility	The combination of speed, acceleration, balance, power, and coordination is demonstrated as an athlete’s ability to move quickly and change directions in the shortest time possible.^38,48^ This definition can also be expanded to include perceptual decision-making elements.^39^
Cardiorespiratory endurance	The maximal ability of the heart, lungs, and muscles to provide the body with oxygen during exercise for an extended period of time.^49^

**Table 2.** Inclusion and exclusion criteria

Inclusion Criteria	Exclusion Criteria
Population	
Human participants, males and females, aged 12-50 years Participants post-ACL-R, primary Must satisfy 2 of 3 following criteria: Athletes (designated by author) Returning to sport or completing RTS testing Tegner Activity Scale score ≥6	Animal models or cadavers Participants post-ACL repair (ie, surgical reattachment of the ACL instead of performing a reconstruction) Participants exclusively post-ACL-R, secondary or complex cases Participants have other significant comorbidities, including musculoskeletal, neurologic, and/or systemic disorders
Intervention	
Speed tests (timed sprints over a set distance, eg, 20 m) Agility tests (timed multidirectional movements through a standardized drill, eg, pro-agility) Strength tests (single or multiple repetition maximums for bilateral closed kinetic chain exercise) Cardiovascular endurance tests (bilateral GXT VO_2_max or field test, eg, beep test)	-
Comparator or Control	
Comparisons of the affected limb to the unaffected limb Comparisons of ACL-R participants to HCG Comparisons to normative testing values	-
Outcomes	
Studies which report APA results Studies which report the rate and level of RTS Studies which report reinjury rates after RTS	-
Timing	
APA occurs between the first 6 months of postsurgical rehabilitation and the 2 years after RTS	APA only occurs <6 months postsurgery or >2 years after RTS
Study Design	
Primary study design (quantitative and mixed methods) with original published data, randomized control trials, pilot studies, case studies, cohort studies, and diagnostic studies	Qualitative studies and not primary study design or original data (conference proceedings or abstracts, editorials, commentaries, opinion-based papers and systematic, scoping, or narrative reviews)

ACL, anterior cruciate ligament; ACL-R ACL reconstruction surgery; APA, athletic performance assessments; GXT, graded exercise test; HCG, healthy control groups; RTS, return to sport; VO_2_max, maximal aerobic capacity.

### Identification and Selection of Studies

In August and September 2021, we searched the following electronic databases: MEDLINE, EMBASE, CINAHL, SPORTDiscus, Scopus, Web of Science, and ProQuest Dissertations and Theses Global. These databases were searched since inception with no language limitations. The final list of systematic search terms is in Appendix 1 (available in the online version of this article). In addition, the reference lists from relevant reviews were screened.

Records obtained from each electronic database were exported into the reference management software Covidence (Veritas Health Innovation, Melbourne, Australia; available at https://www.covidence.org/), where duplicates were removed. A single rater completed the title and abstract screening. Then, 2 raters determined the final study selections by independently performing the full-text review with data extraction. The 2 raters reached a substantial agreement (Cohen’s kappa 87.18%, 0.65; 95% CI, 0.36-0.94). The 2 reviewers’ disagreements on study eligibility were resolved through thorough discussion.

## Results

### Study Selection

**Figure 1.**
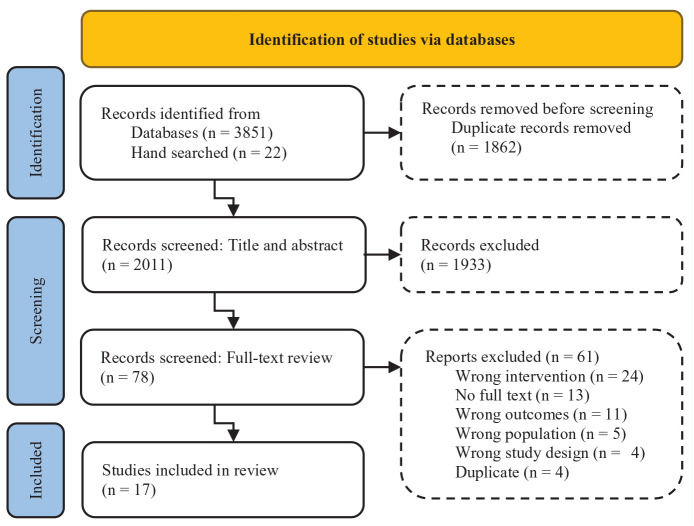
PRISMA-ScR flow diagram. PRISMA, preferred reporting items for systematic reviews and meta-analyses; PRISMA-ScR, PRISMA extension for scoping reviews.

The electronic database search revealed 3873 articles, of which 2011 unique articles proceeded to title and abstract screening, and 78 articles were reviewed in full-text screening. A total of 17 articles met eligibility criteria and were included in the present scoping review. The systematic search and screening results are presented in the PRISMA-ScR flow diagram (Figure 1). Despite searching databases from inception, all eligible publications were published between 2011 and 2021. A total of 24 instances of APA were reported with substantial test selection and protocol heterogeneity. A descriptive analysis of the extracted variables was conducted by categorizing APA by speed, agility, strength, or cardiovascular endurance. Protocols described by the authors helped to distinguish variations among similarly named tests. Further analysis of participant demographics, including sport and level of participation, helped to determine the commonality and applicability of the included APA (Tables 3 and 4).

**Table 3.** APA frequency

Total APA	24
Speed	3
20 m sprint^42^	1
3/4 Court Sprint^29^	1
40-yard dash (36.5 m)^23^	1
Agility	18
3-Cone “L” drill^23,33^	2
5-Cone Agility^13,25^	2
Illinois Agility^6^	1
Lane Agility^29^	1
Long Shuttle^29^	1
Pro-Agility^23,32,33^	3
Reactive shuttle run^29^	1
Shuttle run^20,27^	2
T-Test Agility^16,24,26,32,40^	5
Strength	1
mRM back squat^19^	1
Cardiovascular Endurance	2
Treadmill GXT^14,34^	2

APA, athletic performance assessments; GXT, graded exercise test; mRM = multiple repetition maximum.

**Table 4.** Study characteristics including participant demographics and APA outcome deficits

Author and Study Design	Sport; Level Sex, M/F	APA	APA Outcome Deficits	Rate of RTS	Rate of Reinjury
de Almeida et al^14^ Case-Control Study	Soccer; Professional ACL-R 20/0; HCG 20/0	Treadmill GXT	ACL-R, T1 to T2**; T1, ACL-R < HCG**; T2, ACL-R < HCG**	NR	NR
Blakeney et al^6^ Diagnostic Study	NR; Amateur, Professional ACL-R 287/84; HCG 29/10	Illinois Agility	ACL-R = HCG, ns	NR	NR
Czamara et al^13^ Cohort Study	Tegner 7-8; NR ACL-R 15/15	5-Cone Agility	ns	NR	NR
Dickerson et al^16^ Controlled Laboratory Study	NR; Amateur ACL-R 19/9	T-Test Agility	T1 to T2**	Previous RTS	NR
Horschig et al^19^ Case Report	Football; Amateur ACL-R 1/0	Back Squat; 10RM, 6RM, 3RM	NR	NR	NR
Jang et al^20^ Case Series Study	Soccer, Basketball, Other; NR ACL-R 67/0	Shuttle Run	ns	51/67 (76.12%) RTS	NR
Keller et al^23^ Cohort Study	Football; NFL Draft Eligible ACL-R 98/0; HCG 98/0	40-yard dash	ACL-R = HCG, ns	Previous RTS	NR
		Pro-Agility	ACL-R = HCG, ns		
		3-Cone “L” Drill	ACL-R = HCG, ns		
Kirsch et al^24^ Case-Control Study	Tegner 6.5; NR ACL-R 9/11; HCG 13/12	T-Test Agility	ACL-R < HCG*	NR	NR
Krolikowska^25^ Cohort Study	Tegner 5-8; NR ACL-R 30/0; HCG 30/0	5-Cone Agility	ACL-R (SR) < ACL-R (LR) & HCG**; ACL-R (LR) = HCG, ns	Previous RTS	NR
Kyritsis et al^26^ Cohort Study	Soccer, Handball, Other; Professional ACL-R 158/0	T-Test Agility	ns	Previous RTS	Passed 6 RTS Criteria: 12/116 (10.34%)** Failed ≥ 1 RTS Criteria: 14/42 (33.33%)**
Lee et al^27^ Case Series Study	Tegner 6.5; NR ACL-R 75/0	Shuttle Run	ns	NR	NR
Mehran et al^29^ Cross-Sectional Study	Basketball; NBA Draft Eligible ACL-R 21/0; HCG 21/0	3/4 Court Sprint	ACL-R = HCG, ns	Previous RTS	NR
		Lane Agility	ACL-R = HCG, ns		
		Reactive shuttle run	ACL-R = HCG, ns		
Myer et al^32^ Case-Control Study	Football, Soccer, Basketball, Volleyball; Amateur ACL-R 18; HCG 20 - (M/F NR)	T-Test Agility	ACL-R = HCG, ns; LSI, ns	NR	NR
		Pro-Agility	ACL-R = HCG, ns; LSI, ns		
		Long Shuttle	ACL-R = HCG, ns; LSI, ns		
Nyland et al^33^ Cohort Study	Soccer, Football, Basketball, Other; Amateur ACL-R 83/67	Pro-Agility	LSI, ns	126/150 (84%) RTS	10/150 (6.67%)
		3-Cone “L” Drill	LSI, ns		
Patras et al^34^ Case Series Study	Soccer; Amateur ACL-R 14/0	Treadmill GXT	NR	Previous RTS	NR
Souissi et al^40^ Randomised Control Trial	Soccer, Other; Professional ACL-R 16/0	T-Test Agility	T1 to T2*; T2 FTG > T2 CTG**	NR	NR
Teichmann et al^43^ Cohort Study	Soccer, Other; National ACL-R 16/8	20 m Sprint	T1 to T2*	NR	NR

ACL, anterior cruciate ligament; ACL-R ACL reconstruction surgery; APA, athletic performance assessments; CTG, control training group; F, female; FTG, functional training group; GXT, graded exercise test; HCG, healthy control groups; LR, long rehabilitation; LSI, leg symmetry index; M, male; NBA, National Basketball Association; NFL, National Football League; NR, not reported; ns, not significant; RTS, return to sport; SR, short rehabilitation; T1 to T2, trial 1 to trial 2 or pretest to post-test; *P < 0.05); **P < 0.001.

### Agility

Agility was the most commonly reported APA. A total of 18 agility assessments were used across 15 studies (Tables 3 and 4). T-Test Agility was the most frequently used APA (Table 3). Outcomes of T-Test Agility conducted over multiple timepoints resulted in significant improvements for ACL-R athletes^16,40^; however, ACL-R athletes performed significantly worse than HCG.^24^ When used to assess LSI, T-Test Agility could not determine side-to-side differences.^32^ Similarly, Pro-Agility results improved significantly between trials but did not identify significant LSI.^32,33^ For National Football League (NFL) draft-eligible athletes, Pro-Agility results were comparable between ACL-R and age-, height-, weight-, and position-matched HCG.^23^ The Shuttle Run agility test was found to have a strong correlation (*r* = −0.54, *P* = 0.002) with single-leg vertical jumping - a standard ACL-R RTS test.^27^ The 3-Cone “L” Drill results were comparable between ACL-R and healthy age-, height-, weight-, and position-matched NFL draft-eligible athletes.^23^ The 3-Cone “L” Drill did not determine significant LSI in ALC-R athletes.^33^ For National Basketball Association (NBA) draft-eligible athletes, Lane Agility times were comparable between ACL-R and age-, height-, weight-, and position-matched HCG.^29^ A modified version of the NFL’s Long Shuttle drill did not determine side-to-side asymmetries for ACL-R athletes or the HCG.^32^ The 5-Cone Agility test determined significant differences between 2 different ACL-R rehabilitation protocols,^25^ but found no significant differences related to various surgical procedures.^13^ The Illinois Agility test did not determine any significant difference between ACL-R and HCG athletes.^6^ Illinois Agility test performance was moderately correlated with the total testing battery score (*r* = -0.51, *P* ≤ 0.05).^6^ For NBA draft-eligible athletes, reactive shuttle run times were comparable between ACL-R and age-, height-, weight-, and position-matched HCG.^29^

### Speed

Three studies assessed speed using 3 different APA (Tables 3 and 4). For NFL and NBA draft-eligible collegiate athletes, three-quarter Court Sprint and 40-yard (36.5 m) dash times were comparable between ACL-R and age-, height-, weight-, and position-matched HCG.^23,29^ Malaysian national athletes showed significant improvements in their 20 m sprint results before RTS during their final rehabilitation phase.^42^ The sprint improvement effect size was large for males athletes and moderate for females athletes (*d* = 1.06, *d* = 0.58, respectively; *P* < 0.05).^42^

### Cardiorespiratory Endurance

Two studies included assessments of cardiorespiratory endurance through a treadmill graded exercise test (GXT) maximal aerobic capacity (VO_2_max) APA (Tables 3 and 4). VO_2_max and ventilatory threshold results showed significant improvements in ACL-R athletes after 6 months of rehabilitation compared with their presurgery trial.^14^ The ACL-R measures were still significantly lower than HCG athletes of the same sport and competitive level.^14^ Neuromuscular response of ACL-R athletes during a treadmill GXT was correlated strongly with endurance markers in the unaffected leg (*r* = 0.77, *P* = 0.001) but only moderately in the ACL-R leg (*r* = 0.47, *P* = 0.09).^34^

### Strength

Only 1 study assessed strength through a single APA (Tables 3 and 4). The individual participant demonstrated increases in predicted 1RM back squat across 3 testing trials; however, the significance of the results was not reported.^19^

### Rates of RTS/Reinjury

Two studies (12%) reported rates of RTS, neither of which demonstrated a significant relationship with their respective APA results (Table 4).^20,33^ Only 1 of these studies also reported injury rates, which were also not found to be significantly related to APA.^33^ A second study reporting reinjury rates found a significant decrease in reinjury rates for participants who passed a 6 criterion RTS battery, including T-Test Agility, compared with participants who had not passed all 6 criteria.^26^ However, T-Test Agility alone did not relate directly to RTS.^26^ Six studies (35%) included only ACL-R participants who had RTS before testing. Nine studies (53%) did not report rates of RTS, and 15 studies (88%) did not report reinjury rates.

## Discussion

This scoping review provides evidence that APA has been used in ACL-R RTS research in a limited fashion. Practical recommendations are difficult to elucidate due to a paucity of literature, lack of homogeneity in APA selection, and variability within protocols. Nevertheless, this review may serve as a helpful starting point for stakeholders developing RTS testing procedures. This review sought to explore the relationship between APA and the rate of RTS and reinjury, which were found to be scarcely reported. In addition, this review sought to determine whether ACL-R athletes demonstrated deficits in APA outcomes compared with HCG and whether they might be capable of reducing or eliminating these deficits before RTS. It is evident that APA are valuable in detecting performance deficits after ACL-R, which can improve throughout the rehabilitation process. Currently, APA do not appear capable of detecting LSI deficits. Nevertheless, the potential for ACL-R athletes to improve upon, and even match, the performance of healthy athletes at any level of sports shows promise for their inclusion in RTS testing batteries.

Currently, time from surgery is the most dominant factor for RTS clearance in research, followed by physical measures of strength and power through open-chain isokinetic leg extension or flexion and hop tests.^10^ There has been increasing advocacy for the diversification of RTS testing to improve RTS rates and reduce the likelihood of reinjury.^1,2,10^ The World Congress in Sports Physical Therapy outlines recommendations to guide practitioners when choosing RTS tests, including using a multitest battery, choosing less controlled tests when possible, adding tests with reactive decision-making elements, assessing psychological readiness, and monitoring workload.^2^

APA can be added to current seamlessly RTS testing batteries because they are cost-effective and require minimal equipment. In addition, benchmarks are often obtained easily across many sports, ages, sexes, and levels of competition for healthy and injured athletes due to their frequent use in sports settings. Therefore, it may be possible for practitioners to tailor their RTS testing batteries and benchmarks to their patients by including APA utilized by their team or sport.

Incorporating APA into current ACL-R RTS testing best practices may face barriers to adoption by rehabilitation practitioners due to the limited evidence as a prognostic tool for rates of RTS or reinjury. However, APA have long been used to profile physical abilities for several key performance indicators intended to increase the transfer of training, enhance performance, and reduce the rate of injuries.^36^ Research investigating the association between APA outcomes and general lower-body injuries may help support their inclusion into ACL-R RTP testing batteries until more ACL-R-specific evidence is obtained.

For instance, a systematic review of associations between physical fitness and musculoskeletal injuries demonstrated moderate evidence that slower sprint times were associated with One of the studies included in this review found that rugby players with slow sprint times were approximately 10 times as likely to suffer a lower body injury than their faster counterparts.^17^ The same review did not find an association between agility test performance and rates of musculoskeletal injuries.^15^

For APA of strength, a 150% bodyweight (1.5×BW) back squat is often a benchmark for high-performance athletes.^39^ It is recommended that this be achieved before integrating advanced, high-impact plyometrics due to the high joint and tissue load and subsequent risk of injury.^39^ Male and female collegiate athletes with higher 1RM (1 repetition maximum) back squats were significantly less likely to sustain a lower-body injury than their weaker counterparts (*P* = 0.02 and 0.04, respectively).^12^ The mean relative 1RM back squat for the stronger, uninjured group was 2.2×BW for males and 1.6×BW for females athletes.^12^ These values, obtained from high-level adult athletes, can take years of strength training to achieve. In another study, relative back squat 1RM recommendations for adolescent and youth athletes were 2.0×BW for 16- to 19-year-olds, 1.5×BW for 13- to 15-year-olds, and 0.7×BW for 11- to 12-year-olds, demonstrating the gradual progression to these benchmarks.^22^

Cardiovascular endurance plays a prominent role in endurance sports, but is also a useful measure of fitness for speed-power athletes. Increased cardiovascular endurance has been shown to aid in repeat sprint ability, which is a valued trait for many sports.^21^ Lower cardiovascular endurance has also been linked to a significant increase in the likelihood of injury in adolescent and collegiate athletes (*P* = 0.01).^46,47^ Speed-power athletes with higher cardiovascular endurance have also demonstrated better reaction times.^28^ When athletes are fatigued with maximal cardiovascular endurance work, their movement and skill accuracy decrease, impacting their overall sports performance.^43^

## Limitations

There are major limitations to this scoping review that stakeholders should consider. Many commonly used APA are not represented in this review. It was beyond the scope of this review to assess the validity and reliability of the APAs used. Due to the limited body of literature, many study designs were included, and study quality was not a consideration for exclusion.

## Conclusion

Agility makes up 75% of the APA in the ACL-R RTS literature. APA for speed and cardiovascular endurance make up 12.5% and 8.3%, respectively. Strength measured through bilateral closed kinetic exercise represents only 4.2%. Participants were tested primarily just before or after their RTS. Only 17.6% of studies reported RTS or reinjury rates. Deficits in APA outcomes for ACL-R athletes compared with HCG were common; however, many studies showed significant improvements over time. There is evidence that well-trained ACL-R athletes can match the performance of uninjured athletes in high-level sports.

## Supplemental Material

sj-docx-1-sph-10.1177_19417381221147524 – Supplemental material for Anterior Cruciate Ligament Reconstruction Return-to-Sport Decision-Making: A Scoping Review Click here for additional data file. Supplemental material, sj-docx-1-sph-10.1177_19417381221147524 for Anterior Cruciate Ligament Reconstruction Return-to-Sport Decision-Making: A Scoping Review by Eric Golberg, Mark Sommerfeldt, Adam Pinkoski, Liz Dennett and Lauren Beaupre in Sports Health: A Multidisciplinary Approach
