# Supplementary material for: Anterior Cruciate Ligament Reconstruction Return-to-Sport Decision-Making: A Scoping Review
Source: Sports Health. 2023 Jan 27;16(1):115–23. doi: 10.1177/19417381221147524 (PMC10732109; doi:10.1177/19417381221147524)
Supplement: sj-docx-1-sph-10.1177_19417381221147524 – Supplemental material for Anterior Cruciate Ligament Reconstruction Return-to-Sport Decision-Making: A Scoping Review [file sj-docx-1-sph-10.1177_19417381221147524.docx]

**Appendix 1**

**Ovid MEDLINE(R) ALL 1946 to 2021**

**Date searched: August 30, 2021**

**Results: 463**

1. exp anterior cruciate ligament reconstruction/
2. (Anterior cruciate ligament reconstruction OR ACL Reconstruction OR  ACLR OR ACL-R) OR ((anterior cruciate ligament) adj10 (reconstruct* OR surg*)).mp.
3. Exp Athletic performance/
4. (fitness test OR fitness assess* OR exercise test OR exercise assess* OR athletic performance) OR (SPRINT OR 40 yard dash OR 20 meter OR 30 meter fly) OR (JUMP OR relative power OR vertical jump OR approach jump OR broad jump OR long jump) OR (AGILITY or change of direction OR proagility shuttle test OR pro agility OR 5-10-5 agility OR 3 cone drill OR lane agility OR reactive shuttle test) OR (MAX* STRENGTH OR relative strength OR 1RM OR one rep* max* OR single rep* max* OR multi* rep* max* OR squat OR deadlift OR leg press OR lunge OR split-squat OR power clean OR squat clean OR hip extension OR hip thrust) OR (AEROBIC OR maximal oxygen uptake OR maximal aerobic capacity OR VO2 max OR VO2max OR beep test OR multi* stage fitness test OR pacer test OR bleep test OR Leger 20 meter shuttle run OR yo-yo endurance OR yo-yo intermittent endurance OR yo-yo intermittent recovery OR 30-15 intermittent fitness) OR (ANAEROBIC OR anaerobic power OR anaerobic capacity OR wingate test OR repeat sprint ability OR sprint fatigue).mp.
5. Exp Athletes/
6. Exp Sports/
7. (athlet* OR sport* OR team* OR play* OR ((athlet* OR sport* OR team* OR play*) adj4 (varsity OR university OR collegiate OR intercollegiate OR club OR semi-pro* OR pro* OR professional OR national OR international OR Olympic OR NFL OR NBA OR MLB OR MLS OR CFL OR UEFA OR FIFA OR AFL OR NRL OR WNBA OR NWSL OR NWHL))).mp.
8. ((athlet* OR sport* OR team* OR play*) adj4 (football OR soccer OR basketball OR rugby OR hockey OR skat* OR ringette OR volleyball OR tennis OR gymnastics OR lacrosse OR baseball OR softball OR wrestling OR track OR field OR weightlift* OR runner* OR ski* OR snowboard* )).mp.
9. 1 OR 2
10. 3 OR 4
11. 5 OR 6 OR 7 OR 8
12. 9 AND 10 AND 11

**Embase 1974 to 2021 (OVID interface)**

**Date searched: August 30, 2021**

**Results: 565**

1. exp anterior cruciate ligament reconstruction/
2. (Anterior cruciate ligament reconstruction OR ACL Reconstruction OR  ACLR OR ACL-R) OR ((anterior cruciate ligament) adj10 (reconstruct* OR surg*)).mp.
3. Exp Athletic performance/
4. (fitness test OR fitness assess* OR exercise test OR exercise assess* OR athletic performance) OR (SPRINT OR 40 yard dash OR 20 meter OR 30 meter fly) OR (JUMP OR relative power OR vertical jump OR approach jump OR broad jump OR long jump) OR (AGILITY or change of direction OR proagility shuttle test OR pro agility OR 5-10-5 agility OR 3 cone drill OR lane agility OR reactive shuttle test) OR (MAX* STRENGTH OR relative strength OR 1RM OR one rep* max* OR single rep* max* OR multi* rep* max* OR squat OR deadlift OR leg press OR lunge OR split-squat OR power clean OR squat clean OR hip extension OR hip thrust) OR (AEROBIC OR maximal oxygen uptake OR maximal aerobic capacity OR VO2 max OR VO2max OR beep test OR multi* stage fitness test OR pacer test OR bleep test OR Leger 20 meter shuttle run OR yo-yo endurance OR yo-yo intermittent endurance OR yo-yo intermittent recovery OR 30-15 intermittent fitness) OR (ANAEROBIC OR anaerobic power OR anaerobic capacity OR wingate test OR repeat sprint ability OR sprint fatigue).mp.
5. Exp Athlete/
6. Exp Sport/
7. (athlet* OR sport* OR team* OR play* OR ((athlet* OR sport* OR team* OR play*) adj4 (varsity OR university OR collegiate OR intercollegiate OR club OR semi-pro* OR pro* OR professional OR national OR international OR Olympic OR NFL OR NBA OR MLB OR MLS OR CFL OR UEFA OR FIFA OR AFL OR NRL OR WNBA OR NWSL OR NWHL))).mp.
8. ((athlet* OR sport* OR team* OR play*) adj4 (football OR soccer OR basketball OR rugby OR hockey OR skat* OR ringette OR volleyball OR tennis OR gymnastics OR lacrosse OR baseball OR softball OR wrestling OR track OR field OR weightlift* OR runner* OR ski* OR snowboard* )).mp.
9. 1 OR 2
10. 3 OR 4
11. 5 OR 6 OR 7 OR 8
12. 9 AND 10 AND 11

**CINAHL Plus with Full Text (EBSCOhose interface)**

**Date searched: August 30, 2021**

**Results: 780, 764 after deselecting apply equivalent heading**

1. MH “anterior cruciate ligament reconstruction”
2. (Anterior cruciate ligament reconstruction OR ACL Reconstruction OR  ACLR OR ACL-R) OR ((anterior cruciate ligament) N10 (reconstruct* OR surg*))
3. MH “Athletic performance”
4. (fitness test OR fitness assess* OR exercise test OR exercise assess* OR athletic performance) OR (SPRINT OR 40 yard dash OR 20 meter OR 30 meter fly) OR (JUMP OR relative power OR vertical jump OR approach jump OR broad jump OR long jump) OR (AGILITY or change of direction OR proagility shuttle test OR pro agility OR 5-10-5 agility OR 3 cone drill OR lane agility OR reactive shuttle test) OR (MAX* STRENGTH OR relative strength OR 1RM OR one rep* max* OR single rep* max* OR multi* rep* max* OR squat OR deadlift OR leg press OR lunge OR split-squat OR power clean OR squat clean OR hip extension OR hip thrust) OR (AEROBIC OR maximal oxygen uptake OR maximal aerobic capacity OR VO2 max OR VO2max OR beep test OR multi* stage fitness test OR pacer test OR bleep test OR Leger 20 meter shuttle run OR yo-yo endurance OR yo-yo intermittent endurance OR yo-yo intermittent recovery OR 30-15 intermittent fitness) OR (ANAEROBIC OR anaerobic power OR anaerobic capacity OR wingate test OR repeat sprint ability OR sprint fatigue)
5. MH “Athletes”
6. MH “Sports”
7. (athlet* OR sport* OR team* OR play* OR ((athlet* OR sport* OR team* OR play*) N4 (varsity OR university OR collegiate OR intercollegiate OR club OR semi-pro* OR pro* OR professional OR national OR international OR Olympic OR NFL OR NBA OR MLB OR MLS OR CFL OR UEFA OR FIFA OR AFL OR NRL OR WNBA OR NWSL OR NWHL)))
8. ((athlet* OR sport* OR team* OR play*) N4 (football OR soccer OR basketball OR rugby OR hockey OR skat* OR ringette OR volleyball OR tennis OR gymnastics OR lacrosse OR baseball OR softball OR wrestling OR track OR field OR weightlift* OR runner* OR ski* OR snowboard* ))
9. S1 OR S2
10. S3 OR S4
11. S5 OR S6 OR S7 OR S8
12. S9 AND S10 AND S11

**SPORTDiscus with Full Text (EBSCOhost interface)**

**Date searched: August 30, 2021**

**Results: 1160**

1. DE “anterior cruciate ligament surgery”
2. (Anterior cruciate ligament reconstruction OR ACL Reconstruction OR  ACLR OR ACL-R) OR ((anterior cruciate ligament) N10 (reconstruct* OR surg*))
3. DE “physical fitness testing”
4. (fitness test OR fitness assess* OR exercise test OR exercise assess* OR athletic performance) OR (SPRINT OR 40 yard dash OR 20 meter OR 30 meter fly) OR (JUMP OR relative power OR vertical jump OR approach jump OR broad jump OR long jump) OR (AGILITY or change of direction OR proagility shuttle test OR pro agility OR 5-10-5 agility OR 3 cone drill OR lane agility OR reactive shuttle test) OR (MAX* STRENGTH OR relative strength OR 1RM OR one rep* max* OR single rep* max* OR multi* rep* max* OR squat OR deadlift OR leg press OR lunge OR split-squat OR power clean OR squat clean OR hip extension OR hip thrust) OR (AEROBIC OR maximal oxygen uptake OR maximal aerobic capacity OR VO2 max OR VO2max OR beep test OR multi* stage fitness test OR pacer test OR bleep test OR Leger 20 meter shuttle run OR yo-yo endurance OR yo-yo intermittent endurance OR yo-yo intermittent recovery OR 30-15 intermittent fitness) OR (ANAEROBIC OR anaerobic power OR anaerobic capacity OR wingate test OR repeat sprint ability OR sprint fatigue)
5. DE “Athletes”
6. DE “Sports”
7. (athlet* OR sport* OR team* OR play* OR ((athlet* OR sport* OR team* OR play*) N4 (varsity OR university OR collegiate OR intercollegiate OR club OR semi-pro* OR pro* OR professional OR national OR international OR Olympic OR NFL OR NBA OR MLB OR MLS OR CFL OR UEFA OR FIFA OR AFL OR NRL OR WNBA OR NWSL OR NWHL)))
8. ((athlet* OR sport* OR team* OR play*) N4 (football OR soccer OR basketball OR rugby OR hockey OR skat* OR ringette OR volleyball OR tennis OR gymnastics OR lacrosse OR baseball OR softball OR wrestling OR track OR field OR weightlift* OR runner* OR ski* OR snowboard* ))
9. S1 OR S2
10. S3 OR S4
11. S5 OR S6 OR S7 OR S8
12. S9 AND S10 AND S11

**SCOPUS (Advanced Search)**

**Date searched: September 5, 2021**

**Results: 503**

1. TITLE-ABS-KEY(“anterior cruciate ligament reconstruction”)
2. TITLE-ABS-KEY((“Anterior cruciate ligament reconstruction” OR “ACL Reconstruction” OR  “ACLR” OR “ACL-R”) OR ((“anterior cruciate ligament”) W/10 (“reconstruct*” OR “surg*” )))
3. TITLE-ABS-KEY("athletic performance")
4. TITLE-ABS-KEY((“fitness test” OR “fitness assess*” OR “exercise test” OR “exercise assess*” OR “athletic performance”) OR (“SPRINT” OR “40 yard dash” OR “20 meter” OR “30 meter fly”) OR (“JUMP” OR “relative power” OR “vertical jump” OR “approach jump” OR “broad jump” OR “long jump”) OR (“AGILITY” or “change of direction” OR “proagility shuttle test” OR “pro agility” OR “5-10-5 agility” OR “3 cone drill” OR “lane agility” OR “reactive shuttle test”) OR (“MAX* STRENGTH” OR “relative strength” OR “1RM” OR “one rep* max*” OR “single rep* max*” OR “multi* rep* max*” OR “squat” OR “deadlift” OR “leg press” OR “lunge” OR “split-squat” OR “power clean” OR “squat clean” OR “hip extension” OR “hip thrust”) OR (“AEROBIC” OR “maximal oxygen uptake” OR “maximal aerobic capacity” OR “VO2 max” OR “VO2max” OR “beep test” OR “multi* stage fitness test” OR “pacer test” OR “bleep test” OR “Leger 20 meter shuttle run” OR “yo-yo endurance” OR “yo-yo intermittent endurance” OR “yo-yo intermittent recovery” OR “30-15 intermittent fitness”) OR (“ANAEROBIC” OR “anaerobic power” OR “anaerobic capacity” OR “wingate test” OR “repeat sprint ability” OR “sprint fatigue”))
5. TITLE-ABS-KEY("Athletes")
6. TITLE-ABS-KEY("Sports")
7. TITLE-ABS-KEY((“athlet*” OR “sport*” OR “team*” OR “play*” OR ((“athlet*” OR “sport*” OR “team*” OR “play*”) W/4 (“varsity” OR “university” OR “collegiate” OR “intercollegiate” OR “club” OR “semi-pro*” OR “pro*” OR “professional” OR “national” OR “international” OR “Olympic” OR “NFL” OR “NBA” OR “MLB” OR “MLS” OR “CFL” OR “UEFA” OR “FIFA” OR “AFL” OR “NRL” OR “WNBA” OR “NWSL” OR “NWHL” ))))
8. TITLE-ABS-KEY((“athlet*” OR “sport*” OR “team*” OR “play*”) W/4 (“football” OR “soccer” OR “basketball” OR “rugby” OR “hockey” OR “skat*” OR “ringette” OR “volleyball” OR “tennis” OR “gymnastics” OR “lacrosse” OR “baseball” OR “softball” OR “wrestl*” OR “track” OR “field” OR “weightlift*” OR “runner*” OR “ski*” OR “snowboard*”))
9. 1 OR 2
10. 3 OR 4
11. 5 OR 6 OR 7 OR 8
12. 9 AND 10 AND 11

**Web of Science Core Collection**

**Date searched: September 5, 2021**

**Results:356**

1. TS= (“anterior cruciate ligament reconstruction”)
2. TS= ((“Anterior cruciate ligament reconstruction” OR “ACL Reconstruction” OR  “ACLR” OR “ACL-R”) OR ((“anterior cruciate ligament”) NEAR/10 (“reconstruct*” OR “surg*” )))
3. TS= (“athletic performance”)
4. TS= ((“fitness test” OR “fitness assess*” OR “exercise test” OR “exercise assess*” OR “athletic performance”) OR (“SPRINT” OR “40 yard dash” OR “20 meter” OR “30 meter fly”) OR (“JUMP” OR “relative power” OR “vertical jump” OR “approach jump” OR “broad jump” OR “long jump”) OR (“AGILITY” or “change of direction” OR “proagility shuttle test” OR “pro agility” OR “5-10-5 agility” OR “3 cone drill” OR “lane agility” OR “reactive shuttle test”) OR (“MAX* STRENGTH” OR “relative strength” OR “1RM” OR “one rep* max*” OR “single rep* max*” OR “multi* rep* max*” OR “squat” OR “deadlift” OR “leg press” OR “lunge” OR “split-squat” OR “power clean” OR “squat clean” OR “hip extension” OR “hip thrust”) OR (“AEROBIC” OR “maximal oxygen uptake” OR “maximal aerobic capacity” OR “VO2 max” OR “VO2max” OR “beep test” OR “multi* stage fitness test” OR “pacer test” OR “bleep test” OR “Leger 20 meter shuttle run” OR “yo-yo endurance” OR “yo-yo intermittent endurance” OR “yo-yo intermittent recovery” OR “30-15 intermittent fitness”) OR (“ANAEROBIC” OR “anaerobic power” OR “anaerobic capacity” OR “wingate test” OR “repeat sprint ability” OR “sprint fatigue”))
